# Dynamic transcriptome profiles of postnatal porcine skeletal muscle growth and development

**DOI:** 10.1186/s12863-021-00984-1

**Published:** 2021-09-06

**Authors:** Yanping Wang, Jiying Wang, Hongmei Hu, Huaizhong Wang, Cheng Wang, Haichao Lin, Xueyan Zhao

**Affiliations:** Shandong Provincial Key Laboratory of Animal Disease Control and Breeding, Institute of Animal Science and Veterinary Medicine, Shandong Academy of Agricultural Sciences, Jinan, 250100 Shandong Province China

**Keywords:** Pig, Muscle growth, Differential gene expression, RNA sequencing

## Abstract

**Background:**

Skeletal muscle growth and development are closely associated with the quantity and quality of pork production. We performed a transcriptomic analysis of 12 *Longissimus dorsi* muscle samples from Tibetan piglets at four postnatal stages of 0, 14, 30, and 60 days using RNA sequencing.

**Results:**

According to the pairwise comparisons between the libraries of the muscle samples at the four postnatal stages, a total of 4115 differentially expressed genes (DEGs) were identified in terms of |log_2_(fold change)| ≥ 1 and an adjusted *P* value < 0.01. Short-time series expression miner (STEM) analysis of the DEGs identified eight significantly different expression profiles, which were divided into two clusters based on the expression pattern. DEGs in cluster I displayed a pattern of decreasing to a nadir, and then a rise, and the significantly enriched gene ontology (GO) terms detected using them were involved in multiple processes, of which the cell cycle, immunocyte activation and proliferation, as well as actin cytoskeleton organization, were the top three overrepresented processes based on the GO terms functional classification. DEGs in cluster II displayed a pattern of increasing to a peak, then declining, which mainly contributed to protein metabolism. Furthermore, besides the pathways related to immune system, a few diseases, and protein metabolism, the DEGs in clusters I and II were significantly enriched in pathways related to muscle growth and development, such as the Rap1, PI3K-Akt, AMPK, and mTOR signaling pathways.

**Conclusions:**

This study revealed GO terms and pathways that could affect the postnatal muscle growth and development in piglets. In addition, this study provides crucial information concerning the molecular mechanisms of muscle growth and development as well as an overview of the piglet transcriptome dynamics throughout the postnatal period in terms of growth and development.

**Supplementary Information:**

The online version contains supplementary material available at 10.1186/s12863-021-00984-1.

## Background

As the growth and development of skeletal muscle is closely associated with meat quality, carcass characteristics, and growth rates in pigs, it has garnered widespread attention. A major component of the skeletal muscle tissue is constituent myofibers, the formation process of which is termed myogenesis that occurs in the stages of prenatal and postnatal growth, development, and regeneration. Muscle fibers are derived from myoblasts, which proliferate, fuse to form myotubes, and finally differentiate into myofibers [1]. Prenatal myogenesis includes primary and secondary myogenesis, which originate from the embryonic and fetal myoblasts that occur between approximately 35–60 days and 55–90 days of gestation, respectively [2]. The two phases mainly determine the total number of muscle fibers (TNF), which are mostly fixed at birth. A third generation of fibers, which also results in an increase in TNF in pigs, has been observed around birth [3–5]. However, the quantitative contribution of tertiary fibers to muscle growth is thought to be very low. Postnatal growth of skeletal muscle is mainly based on the increase in length and girth of myofibers.

Recently, many studies have explored the molecular mechanisms underlying skeletal muscle growth and development in pigs, in which several candidate genes have been found to play important roles in these processes, such as the *MRF* gene families [6–8], *MEF2* [9, 10], and *MSTN* [11, 12]. Among these, *IGF2* is considered as one major gene, and its regulatory mutation can cause a major quantitative trait locus (QTL) effect on muscle growth in pigs [13, 14]. Additionally, several studies have used RNA sequencing (RNA-seq) to elucidate the gene expression patterns during porcine muscle development and growth. McDaneld et al. [15] explored the microRNA (miRNA) profiles of the skeletal muscle during three stages of fetal development (60-, 90-, and 105-day-old fetuses), day-old neonate, and adult. Siengdee et al. [16] conducted a comparative study to explore the muscle miRNA expression profiles and clarify the breed-associated regulation of miRNAs in Landrace and Pietrain pig breeds. Furthermore, the mRNA transcriptome profiles of skeletal muscle tissue have been compared across different developmental stages, including the prenatal [17] and postnatal [18] between western pig breeds, and embryonic to postnatal periods between Chinese indigenous pigs and western pigs [9, 19]. Meanwhile, integrated analysis revealed the miRNA–mRNA paired expression profiles during skeletal muscle development [20, 21]. However, previous transcriptome analysis of porcine skeletal muscle samples concentrated on identifying the breed-specific genes that affect muscle development by comparing two breeds. Few studies have systematically examined the differences in transcriptome profiles at different developmental stages in piglets, although dynamic transcript analyses from the embryonic to postnatal periods have been reported in sheep [22] and chickens [23].

Tibetan pigs, one of the Chinese indigenous pig breeds, is typically distributed in the Qinghai–Tibet Plateau [24]. As a miniature pig breed, Tibetan pigs are increasingly used as animal models for medical studies [25, 26]. As mentioned above, the postnatal period, especially the neonatal period, is the most critical stage of skeletal muscle growth and development. Hence, to systematically characterize the gene expression patterns associated with postnatal muscle growth and development, we carried out a transcriptomic analysis of skeletal muscle in Tibetan piglets at four stages, 0, 14, 30, and 60 days of age. Our study has highlighted the possible genetic mechanisms of postnatal muscle growth and development in pigs, as well as the possible molecular processes that are of agricultural and medical importance.

## Results

### RNA-seq of *longissimus dorsi* (*LD*) muscle samples

To systematically identify the expressed mRNA and their spatiotemporal expression profiles during skeletal muscle growth and development in pigs, cDNA libraries were constructed from *LD* muscle samples of 12 Tibetan pigs at 0, 14, 30, and 60 days, representing the four important developmental stages: D0 (No. D0_1, D0_2, D0_3), D14 (No. 14D_1, 14D_2, 14D_3), M1 (No. 1M_1, 1M_2, 1M_3), and M2 (No. 2M_1, 2M _2, 2M _3). In total, an average of 66.40 million raw reads were obtained in each sample. After filtering out 2.23% of the reads, approximately 29.19 Gb of high-quality data per stage were obtained (Table S1). Of these, 92.32 to 93.88% of the clean reads were mapped to the porcine reference (*Sscrofa* 11.1) genome, with 89.13% that were uniquely mapped (Table S1).

### Identifying the differentially expressed genes (DEGs)

**Fig. 1:**
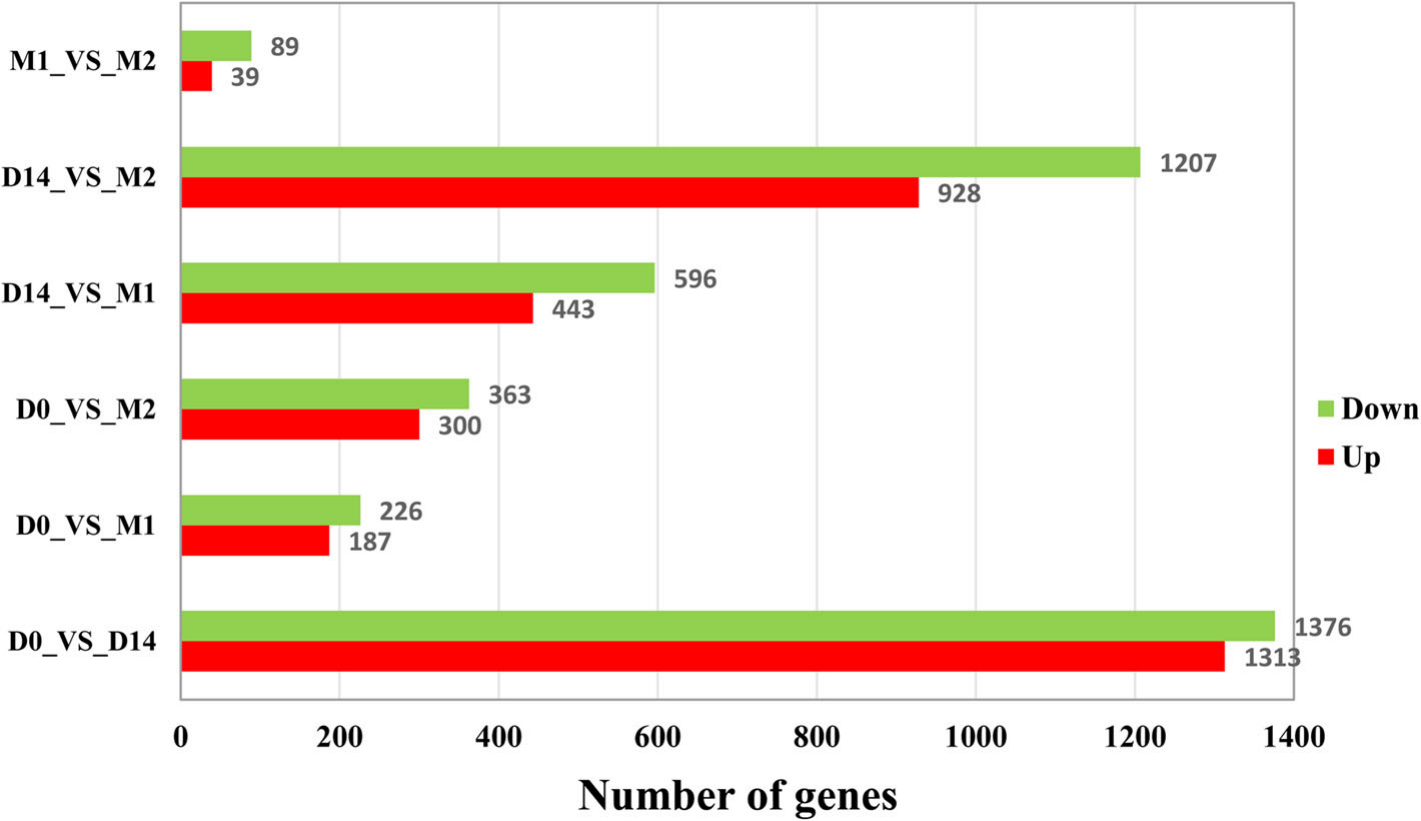
The number of upregulated and downregulated differentially expressed genes (DEGs) in the porcine *Longissimus dorsi* muscle at four developmental stages through pairwise comparisons

**Fig. 2:**
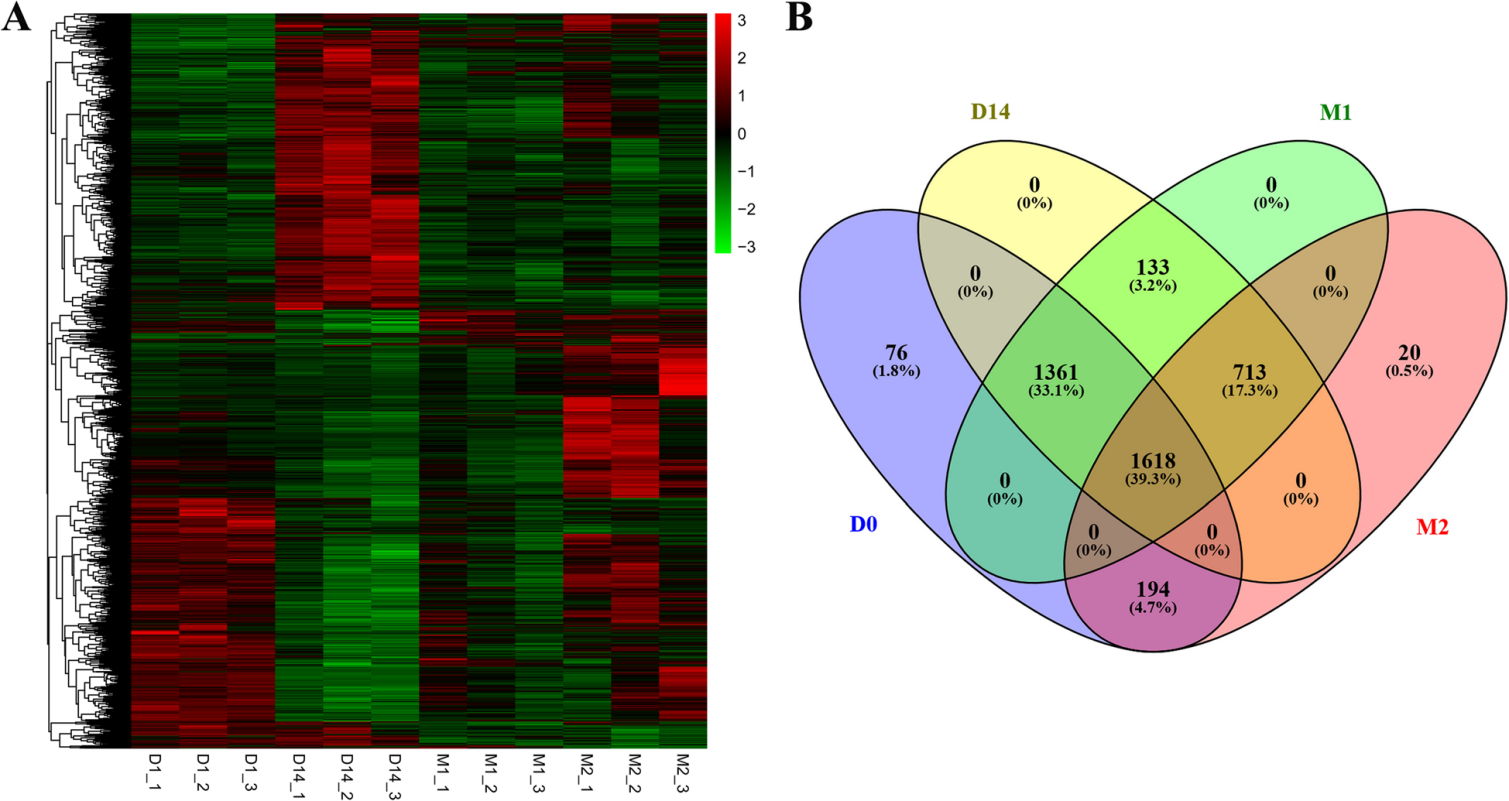
The differentially expressed genes (DEGs) identified by the pairwise comparisons between the libraries of the *Longissimus dorsi* muscle at four developmental stages. (**A**) Heatmap of the DEGs. (**B**) Venn diagram of the identified DEGs at 0, 14, 30, and 60 days of age. D0, D14, M1, and M2 represent 0, 14, 30, and 60 days of age, respectively

As a result of the pairwise comparisons between the libraries of *LD* muscle samples at the four developmental stages, 4115 genes were detected in terms of |log_2_(fold change)| ≥ 1 and an adjusted *P* value < 0.01 (Fig. 1, Table S2). A total of 2689, 413, and 663 DEGs in D0 were found relative to D14, M1, and M2, respectively. In addition, 1039, 2135, and 128 DEGs were detected in D14 relative to M1, in D14 relative to M2, and in M1 relative to M2, respectively (Fig. 1). The most changes in the transcription occurred between D0 and D14 with a similar number of up- and down-regulated genes. The cluster heatmaps of the DEGs can be found in Fig. 2A. In addition, the expression pattern of the DEGs clearly varied across the growth stages. To further understand the relationship among the DEGs, a Venn diagram was generated using 3249, 3825, 1463, and 2545 DEGs that were identified at D0, D14, M1, and M2, respectively (Fig. 2B). Among these genes, there were no stage-specific expressed DEGs, and 775 DEGs were found to be expressed in all four stages (Table S3).

### STEM analysis of the DEGs expression profiles

**Fig. 3:**
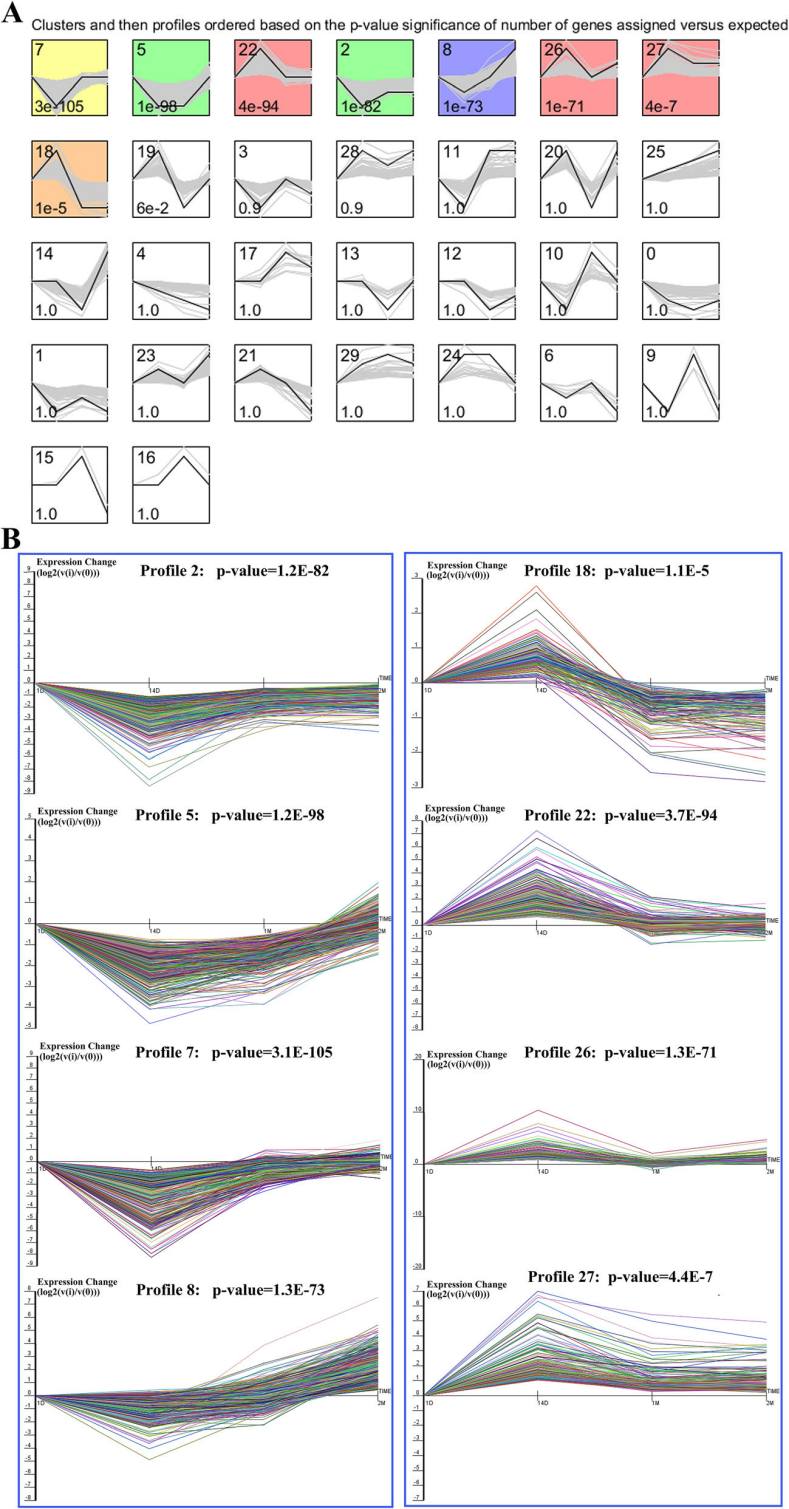
Short-time series expression miner analysis of the expression profiles of the differentially expressed genes (DEGs). (**A**) Each box corresponds to an expression profile type, where only the colored profiles are statistically significant. The upper-left and upper-right numbers in each box indicate the order of profiles and the *P* values, respectively. (**B**) The eight significant clusters of the DEG profiles across all four stages. The profiles 2, 5, 7, and 8 show a decrease to a nadir and then a rise, while profiles 18, 22, 26, and 27 show an increase to a peak, then a decline

Since the transcriptome data was derived from the pigs at four growth time points, STEM was performed to visualize the expression patterns using the 4115 DEGs. Figure 3A shows 30 boxes that represent different module temporal expression profiles. The order of the profiles was based on the significance of the clustering profiles, from the lowest to the highest *P* values. Eight colored profiles, containing a total of 3036 DEGs, included a statistically significant number of genes (*P* < 0.05, Table S4). Of these, profiles 2, 5, 7, and 8 showed a decrease to a nadir, and then a rise, while profiles 18, 22, 26, and 27 showed an increase to a peak, then a decline (Fig. 3B). Therefore, based on the expression pattern, the DEGs were divided into two classes with cluster I that consisted of profiles 2, 5, 7, and 8 with a total of 1752 DEGs as well as cluster II that consisted of profiles 18, 22, 26, and 27 with a total of 1284 DEGs.

### Functional enrichment analysis

**Fig. 4:**
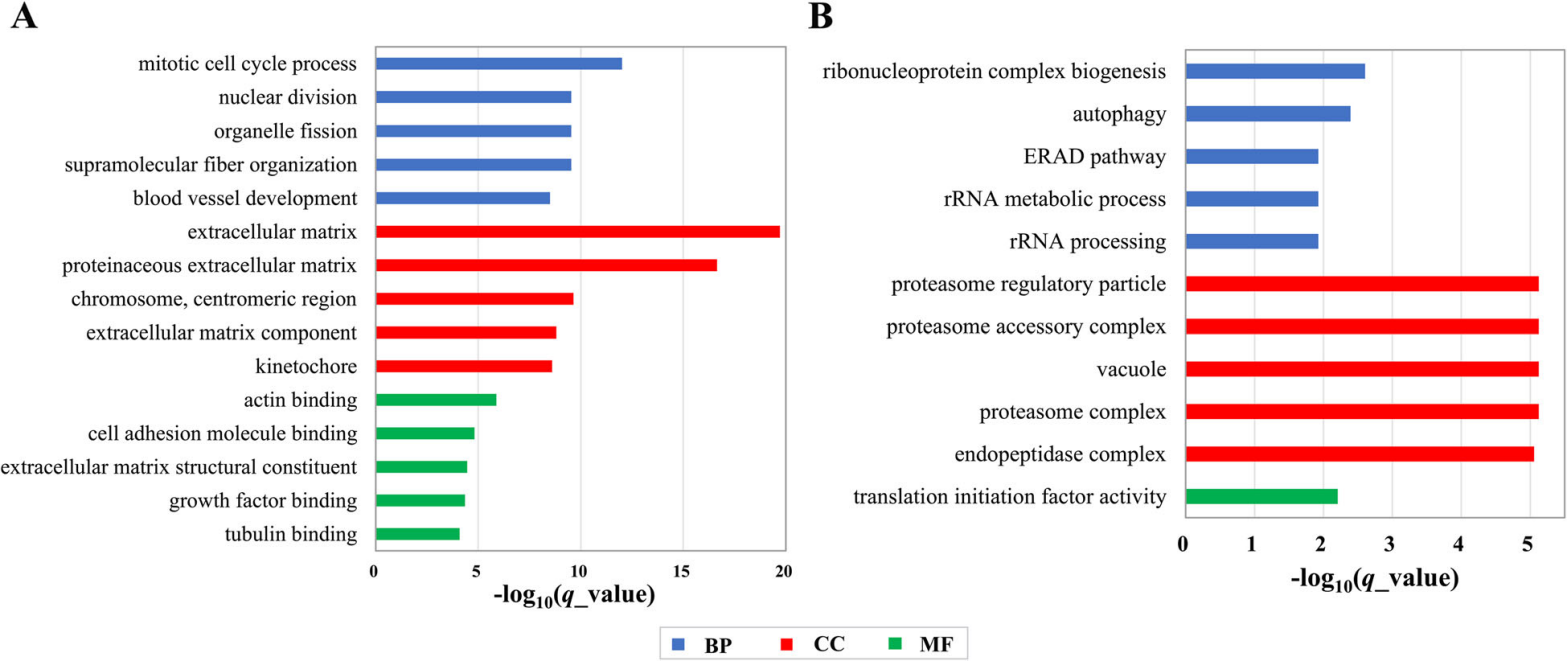
The top five significantly enriched Gene Ontology terms in (**A**) cluster I (profiles 2, 5, 7, and 8) and (**B**) cluster II (profiles 18, 22, 26, and 27)

**Fig. 5:**
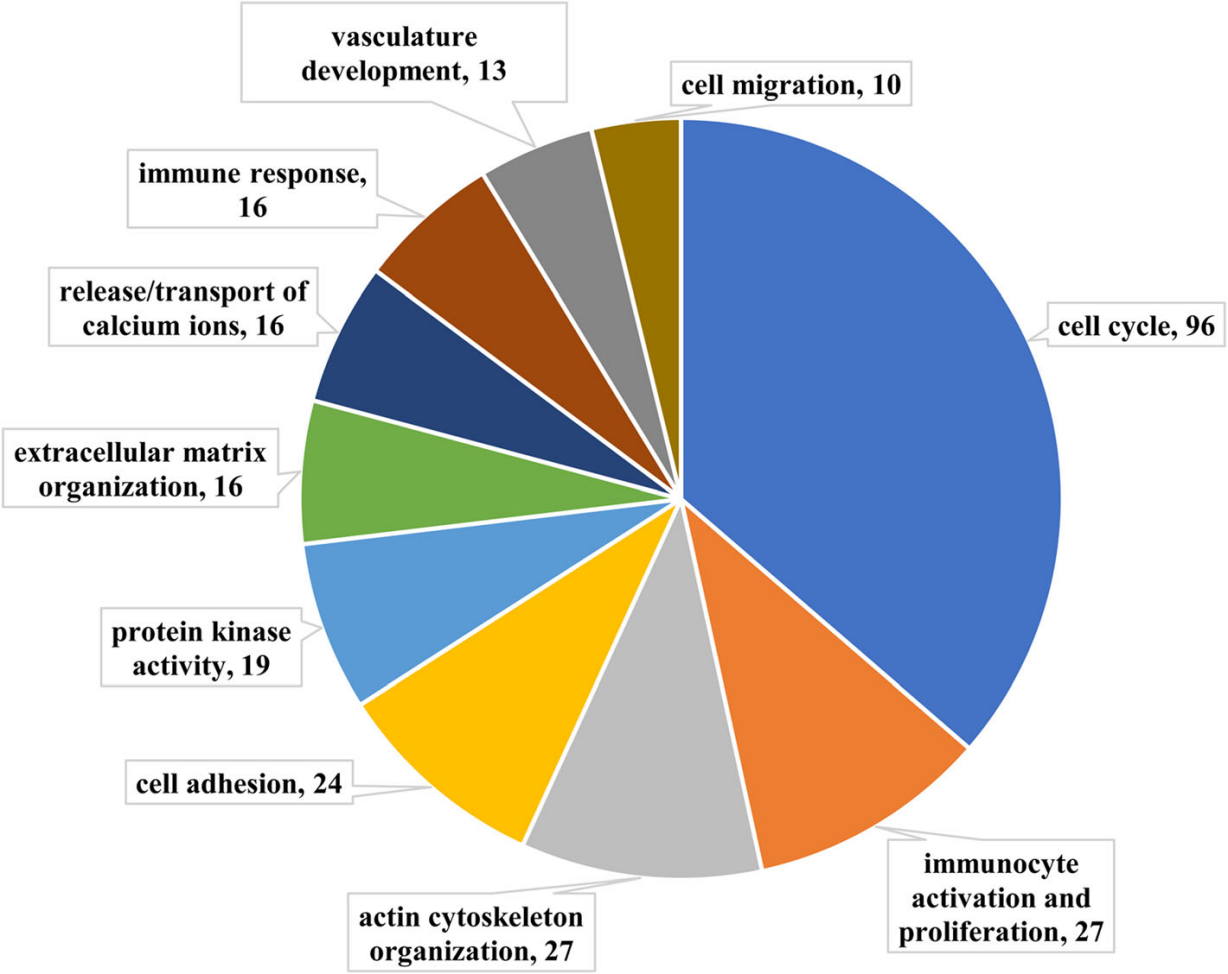
The top ten processes of the significantly enriched Gene Ontology terms of cluster I

To facilitate the biological interpretation of DEGs in clusters I and II, functional enrichment analysis was performed. Figure 4 shows the top five significantly enriched GO terms in terms of biological process (BP), molecular function (MF), and cellular component (CC) categories. Using the genes in cluster I (profiles 2, 5, 7, and 8; Table S5), 432 significantly enriched GO terms, including 348, 65, and 19, belonging to the BP, MF, and CC categories, respectively, were detected after filtering with respect to the corrected *P* value (*q* value) < 0.05. These significantly enriched GO terms were classified according to their function, and Fig. 5 summarizes the top ten processes. The process with the most GO terms was related to cell cycle. In addition to the cell cycle, the processes related to immunocyte activation and proliferation, actin cytoskeleton organization, cell adhesion, protein kinase activity, extracellular matrix organization, release/transport of calcium ions, immune response, vasculature development, and cell migration contained 61.11% of the significantly enriched GO terms. Within the BP category, the top five significantly enriched GO terms were mitotic cell cycle process (*q* = 9.74E-13), supramolecular fiber organization (*q* = 2.93E-10), organelle fission (*q* = 2.93E-10), nuclear division (*q* = 2.93E-10), and blood vessel development (*q* = 3.19E-09). In the case of the CC and MF categories, the most abundant GO term in each category was the extracellular matrix (*q* = 1.95E-20) and actin binding (*q* = 1.31E-06), respectively (Fig. 4A).

Furthermore, the genes in cluster II (profiles 18, 22, 26 and 27) were overrepresented in 52 GO terms, where 24, 27 and one were under the BP, CC, and MF, respectively, which mostly contributed to protein metabolism (Table S5). GO terms related to protein synthesis of protein metabolism were involved in translational initiation, rRNA processing, and ribosome biogenesis. Besides these GO terms, the GO terms involving the proteasome and lysosome, which also played an important role in proteolysis, were also detected. The top overrepresented GO terms included the ribonucleoprotein complex biogenesis (*q* = 2.49E-03), the proteasome regulatory particle (*q* = 7.51E-06), and the translation initiation factor activity (*q* = 6.21E-03) in the BP, MF, and CC categories, respectively (Fig. 4B).

**Fig. 6:**
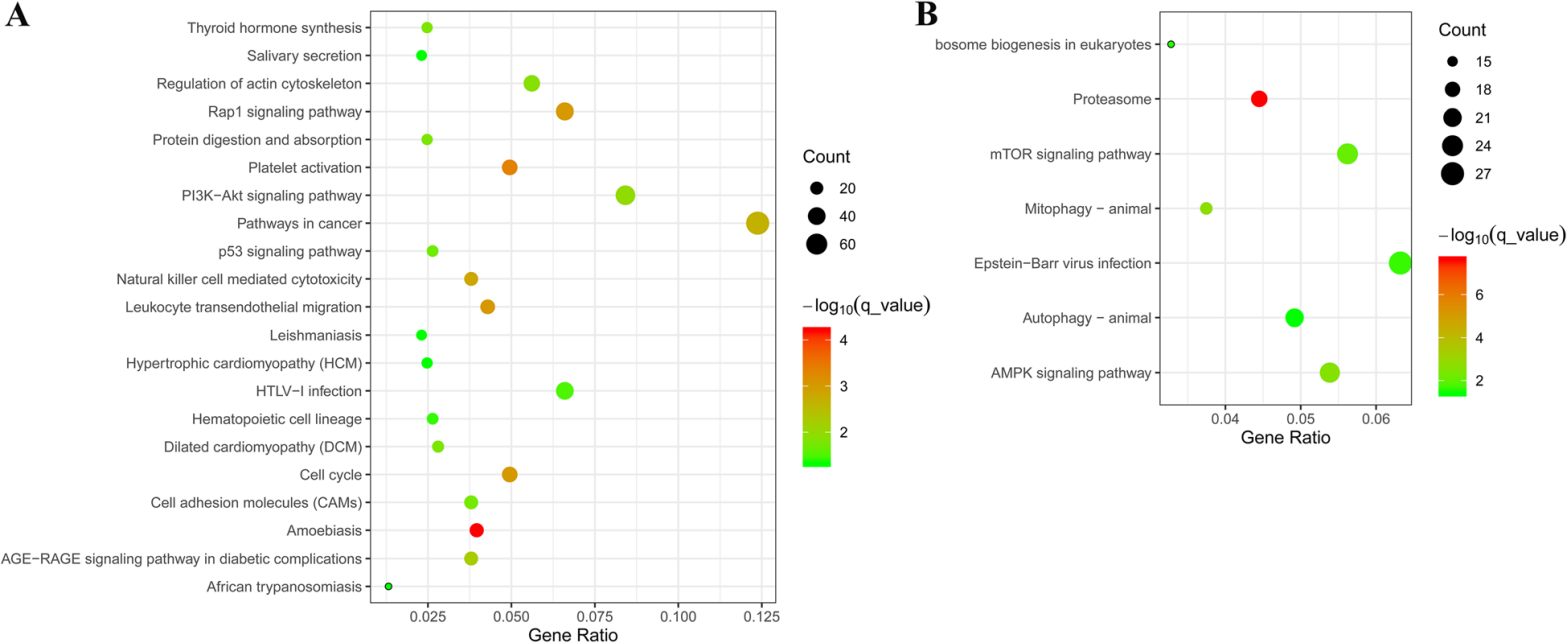
A bubble plot of the significantly enriched pathways of (**A**) cluster I (profiles 2, 5, 7, and 8) and (**B**) cluster II (profiles 18, 22, 26, and 27). The bubble color and size correspond to the *q* value and the gene number enriched in the pathways, respectively

When using DEGs in cluster I, 21 pathways were significantly enriched (*q* < 0.05). Approximately half of these pathways were involved in the immune system and a few diseases, such as amebiasis (*q* = 6.26E-05), platelet activation (*q* = 4.54E-04), and leukocyte transendothelial migration (*q* = 8.58E-04), which were the top three overrepresented pathways. Other pathways related to muscle growth and development were also found, such as the Rap1 signaling pathway (*q* = 9.20E-04), the cell cycle (*q* = 9.33E-04), the PI3K-Akt signaling pathway (*q* = 1.04E-02), and the regulation of the actin cytoskeleton (*q* = 1.32E-02, Fig. 6A). In cluster II, the DEGs were significantly enriched in seven pathways (*q* < 0.05, Fig. 6B). Among these, the proteasome (*q* = 2.36E-08) that participates in proteolysis was the most significantly enriched, and the APMK (*q* = 2.70E-03) and mTOR signaling pathway (*q* = 9.05E-03) were reported to be closely related to muscle growth and development (Fig. 6B).

## Discussion

To understand the dynamics of muscle transcriptome during the first few weeks of pig postnatal period, we used RNA-seq to explore the expression differences at four developmental stages of piglets from D0 to M2. The most expression changes were observed during the neonatal period (D0 relative to D14), where 2689 DEGs were identified. The results were in agreement with those of previous studies, in which the number of DEGs at 0–1 week was the highest during the postnatal stages (0, 1, 3, and 5 weeks) in both Tongcheng and Yorkshire pigs [19]. Compared to the other stages during the postnatal period, these changes at the transcriptional level were likely resulted from the simultaneous hyperplasia and hypertrophy of the myofibers during the neonatal stage of the piglets.

To investigate the dynamic genetic changes starting from newborn to two months old, STEM analysis was performed using the transcriptomic data of the *LD* muscle samples at the four time points. The STEM analysis provides a new approach to cluster, compare and visualize gene expression data from short-time series RNA-seq or microarray experiments [27], which has been widely used in studying the dynamic transcriptomic changes during development in pigs [28–30]. After adjusting a series of parameters, we found that setting the maximum number of model profiles to 2 and retaining the default values of the other parameters were optimal, as eight significantly different expression profiles were found to contain most of the DEGs. Based on the temporal expression pattern, the eight profiles were divided into two clusters. Since genes with similar expression patterns may participate in similar biological processes [31], the functional enrichment analysis of genes in the same cluster could be helpful in revealing the biological processes involved in skeletal muscle development.

The large postnatal increase in muscle mass is accompanied by the hypertrophy of the existing fibers due to the fusion of satellite cells (SCs). SCs proliferate actively and add nuclei to the muscle fibers in the neonate [32]. However, their proliferation is at a decreasingly slower rate, and they are mitotically quiescent in the adult and only become activated in response to injury. Maintaining SCs in an undifferentiated and quiescent state, while protecting SCs from death, require the involvement of cell to cell adhesion [33]. Hence, the functional enrichment analysis of the DEGs in cluster I demonstrated that GO terms and pathways that are related to the cell cycle and adhesion were over-represented (Table S5; Fig. 6A).

In addition, DEGs in cluster I were also significantly enriched in GO terms that are involved in muscle formation, including in the actin cytoskeleton organization and in the extracellular matrix. Under the regulation of a suite of actin-binding proteins, the actin molecules polymerize into actin filaments [34]. The actin filaments, with their accessory and regulatory proteins, comprise the actin cytoskeleton, which is essential for forming and maintaining the shape and structure of cells [35]. In muscle cells, the actin cytoskeleton also plays an important role in the force-generating machinery by generating the pushing forces through the coordinated polymerization of multiple actin filaments as well as the pulling forces through sliding the actin filaments along the bipolar filaments of myosin II [36, 37]. Moreover, the skeletal muscle fibers are surrounded by the extracellular matrix, which regulates the muscle development through interactions of the extracellular matrix molecules with one another, with growth factors, and through the cell-extracellular matrix signal transduction pathways [38]. Furthermore, the GO terms related to the release and transport of calcium ions were overrepresented when using the genes in cluster I. Calcium ion, which acts as the main regulatory and signaling molecule of the muscle, plays a crucial role in muscle function and plasticity by involving in the relaxation after the twitch, maintaining the structural integrity of the muscle fiber, and regulating energy metabolism [39].

Compared to the other stages of the postnatal development, the growth rate of muscle is greater in the neonatal period. To gain a rapid growth and increase in muscle mass, proteins deposition is more rapid in the skeletal muscle tissue than in other tissues. Moreover, the rate of protein synthesis, which is determined by the number of ribosomes in the muscle and their efficiency in translating mRNA into protein [40], is rapid during early development, but it decreases sharply with age [41]. The GO terms related to protein synthesis, such as the ribonucleoprotein complex biogenesis, rRNA metabolic process, translational initiation, and mRNA metabolic process, were significantly enriched when using the DEGs in cluster II (Table S5). In addition, the high rate of protein deposition is issued from the rate of protein synthesis being greater than that of the protein degradation. Therefore, the GO terms related to the proteasome and lysosome, such as the ubiquitin-dependent protein catabolic process, the proteasomal protein catabolic process, and the lysosome, which are important proteolytic systems in the skeletal muscle, were also overrepresented (Table S5). Similar to protein synthesis, protein degradation is also elevated during the neonatal period, however, the rate of it was modest and the changes with age were quite small [42]. That was likely why the genes in cluster II exhibited a “˄” shape of an expression pattern with the highest expression at day 14.

Furthermore, the pathway enrichment analysis provides a good method for understanding the biological functions of genes. In our study, DEGs in clusters I and II were used to perform the Kyoto Encyclopedia of Genes and Genomes (KEGG) pathway analysis for disclosing their putative functions. Besides pathways related to the processes including immune system, a few diseases, and protein metabolism, some pathways related to muscle growth and development including Rap1 signaling, PI3K-Akt signaling, AMPK signaling, and mTOR signaling pathways were also found (Fig. 6). The heatmap of 114 DEGs in these pathways showed that the DEGs could be divided into two groups with 76 and 38 genes respectively, based on the expression pattern of DEGs in cluster I and II detected by STEM (Fig. S1). Rap1, a ubiquitous protein that belongs to Ras family, can regulate diverse physiological responses, such as cell growth, cell adhesion, and cytoskeleton remodeling. Previous studies indicated that Rap1 accumulates during muscle cell differentiation [43], however, one of its isoforms, Rap1A, can inhibit myogenic differentiation by affecting the intracellular degradation [44]. Moreover, the Rap1 protein participates in the autophagy process, which is responsible for maintaining homeostasis during skeletal differentiation and growth [45]. In the case of the PI3K-Akt pathway, its activation could induce hypertrophy of the skeletal muscle by directly inducing the expression of IGF-1 [46], which is also a DEG in cluster I. IGF-1/PI3K/Akt can dominantly inhibit the effects of myostatin, which blocks the differentiation of myoblasts from myotubes [47]. Furthermore, IGF-1 could activate mTOR, which appears to have a central function in integrating different types of growth signals, resulting in protein synthesis of the muscle hypertrophy [48]. Previous studies have also found that Akt/mTOR signaling by IGF-1 can regulate the hypertrophy of skeletal muscle and prevent muscle atrophy [49, 50]. Besides Akt, the activation of mTOR could also be regulated by AMPK for protein synthesis, where AMPK is considered an energy sensor that modulates both glucose uptake and fatty acid oxidation in skeletal muscle [51]. Knockout analysis has confirmed that AMPK promotes myogenesis through a mechanism mediated by AMPKα1, which is one of catalytic α subunits [52].

## Conclusions

In the present study, we provided insight into the temporal expression profiles of DEGs during the skeletal muscle development at four pig postnatal stages of 0, 14, 30, and 60 days using RNA-Seq. We identified a total of 4115 DEGs, which mainly exhibited two significantly different expression patterns and were divided into two clusters. Functional enrichment analysis indicated that genes in cluster I were involved in multiple processes, where the cell cycle, immunocyte activation and proliferation as well as actin cytoskeleton organization were overrepresented. Genes in cluster II mainly participated in GO terms related to protein metabolism. Furthermore, DEGs of the two clusters were enriched in pathways involved in muscle growth and development, such as Rap1, PI3K-Akt, AMPK, and mTOR signaling pathways. In summary, our study could contribute to the understanding of the regulatory mechanisms underlying muscle growth and development in the postnatal stage of piglets.

## Methods

### Animal and sample preparation

Sampled Tibetan pigs were raised together on the breeding farm called Dongsan Pig Breeding Co. Ltd. in Jining, Shandong province, China. The Tibetan population was a closed nucleus breeding system with about 80 sows and 10 boars. Twelve pigs from three sows without sib and half-sib relationships were used in the study. They were normally developed, vital, and without visible defects. At each developmental stage, D0 (just after birth), D14 (14 days after birth), M1 (30 days old, two days after weaning), and M2 (60 days old), three piglets, i.e. one pig from each sow, were selected with one female and two male, or two female and one male, and the average body weight of the pigs at the four stages were 1.07, 2.43, 4.60, and 7.15 kg, respectively (Table S6). Moreover, the male pigs used in this study were not castrated. Three piglets at the same stages were anaesthetized with intravenous injections of 2% pentobarbital sodium (25 mg/kg), and subsequently bled in one batch. LD muscle samples around the fourth-last thoracic vertebrae were sampled and placed in tubes with RNAlater Stabilization Solution (Thermo Fisher, Waltham, MA, USA). Harmless disposal was implemented for the dead piglets followed the procedures of the Institutional Animal Care and Use Committee of the Institute of Animal Science and Veterinary Medicine at the Shandong Academy of Agricultural Sciences (permit number: IACC20060101) after sample collection. At the D0 stage, three newborn piglets were immediately sent to the laboratory for sample collection, without receiving colostrum or any food. The other piglets were kept with sows in the farrowing houses until weaning at 28 days of age, and transferred to nursery houses after weaning. From the 7th day of age to weaning, besides sucking milk, the piglets could get water and nursery formulated diets ad libitum, which were formulated based on the nutritional requirement of piglets. After weaning, the pigs were fed twice daily by nursery formulated diets with free access to water.

### RNA extraction, library construction and sequencing

Total RNA was extracted from the 12 samples using the TRIzol reagent (Invitrogen, Life Technologies, USA) according to the manufacturer’s instructions. The RNA purity was determined using a NanoPhotometer® spectrophotometer (IMPLEN, CA, USA), while the integrity was evaluated using 1% gel electrophoresis and checked by the RNA integrity number (RIN) value measured by the RNA Nano 6000 Assay Kit of the Bioanalyzer 2100 (Agilent Technologies, CA, USA). The RINs of the samples varied between 8.0 to 8.6, where 3 μg of RNA with RIN values greater than 8.0 were used as input material for the RNA library construction. The RNA libraries of the pigs were generated using the NEBNext® Ultra™ RNA Library Prep Kit for Illumina® (NEB, USA) following the manufacturer’ s recommendations. The Poly A mRNA isolation was performed using the poly T oligo-attached magnetic beads, where the sequencing of the libraries was performed using an Illumina HISeq platform (Illumina, Inc., CA, USA) and 150 bp paired-end reads were generated.

### RNA-Seq reads mapping and DEG analysis

The clean data was obtained by filtering the reads with an adapter and poly-N as well as low-quality reads. The HISAT2 software [53] was used for mapping the clean reads to the *S. scrofa* reference genome (*Sscrofa* 11.1) using the annotation database Ensembl Genome Browser v95 [54]. In addition, the number of reads mapped to each gene was calculated using the HTSeq software [55], where the fragments per kilobase million (FPKM) of each gene were measured based on the length of the gene and read count that was mapped to the gene. Moreover, DESeq, using a negative binomial distribution model, was performed to select the DEGs using the read count data [56]. Finally, an adjusted *P* value (*q* value) was calculated using Benjamini and Hochberg’s approach for controlling the false discovery rate, where genes with |log_2_(fold change)| ≥ 1 and a *q* value < 0.01 were considered as DEGs.

### Time series expression profile clustering

The non-parametric clustering algorithm of STEM was performed to cluster and visualize the expression patterns of DEGs on the basis of the FPKM values. The maximum unit change in the model profiles between the time points was set to 2, which is the default value, while the maximum number of model profiles was adjusted to 30. The other parameters in the STEM were set at the default values. In addition, the expression profiles of the DEGs were clustered based on their log_2_(FPKM values) and their correlation coefficients. Moreover, the statistical significance of each profile was calculated based on the number of genes assigned to a profile relative to the expected number of genes [27], where the multiple comparisons were corrected using the Bonferroni correction at *P* < 0.05.

### Functional enrichment analysis

To further understand the putative function of the DEGs, GO and KEGG pathway enrichment analyses were conducted using the R package clusterProfiler [57]. The GO terms and pathways with *q* value (adjusted *P* value by Benjamini and Hochberg’s method) < 0.05 were considered to be significantly enriched ones.

## Supplementary Information

**Additional file 1: Table S1.** Summary of the sequencing data of the 12 Tibetan pigs.

**Additional file 2: Table S2.** List of the identified differentially expressed genes (DEGs) from the pairwise comparisons.

**Additional file 3: Table S3.** The differentially expressed genes (DEGs) that were expressed in all four stages.

**Additional file 4: Table S4.** The differentially expressed genes (DEGs) in the significantly different expression profiles.

**Additional file 5: Table S5.** The significantly enriched Gene Ontology terms (*q* < 0.05) of the differentially expressed genes (DEGs) in clusters I and II.

**Additional file 6: Table S6.** Phenotypic information of the 12 Tibetan pigs.

**Additional file 7: Fig. S1.** Heatmap of the DEGs in the pathways related to muscle growth and development including Rap1 signaling, PI3K-Akt signaling, AMPK signaling, and mTOR signaling pathways.

## Data Availability

The generated data in this study has been included in the main article and its supplementary files. The raw RNA sequencing data was deposited in the National Center for Biotechnology Information Sequence Read Archive with accession number PRJNA527944 (available online).

## Abbreviations

QTL: Quantitative trait locus
RNA-seq: RNA sequencing
TNF: Total number of muscle fibers
DEGs: Differentially expressed genes
FPKM: Fragments per kilobase million
GE: Gene Ontology
*LD*: *Longissimus dorsi*
KEGG: Kyoto Encyclopedia of Genes and Genomes
SCs: Satellite cells
STEM: Short-time series expression miner
BP: Biological process
MF: Molecular function
CC: Cellular component
