# Supplementary figures and images for: Dynamic transcriptome profiles of postnatal porcine skeletal muscle growth and development
Source: BMC Genom Data. 2021 Sep 6;22:32. doi: 10.1186/s12863-021-00984-1 (PMC8419915; doi:10.1186/s12863-021-00984-1)

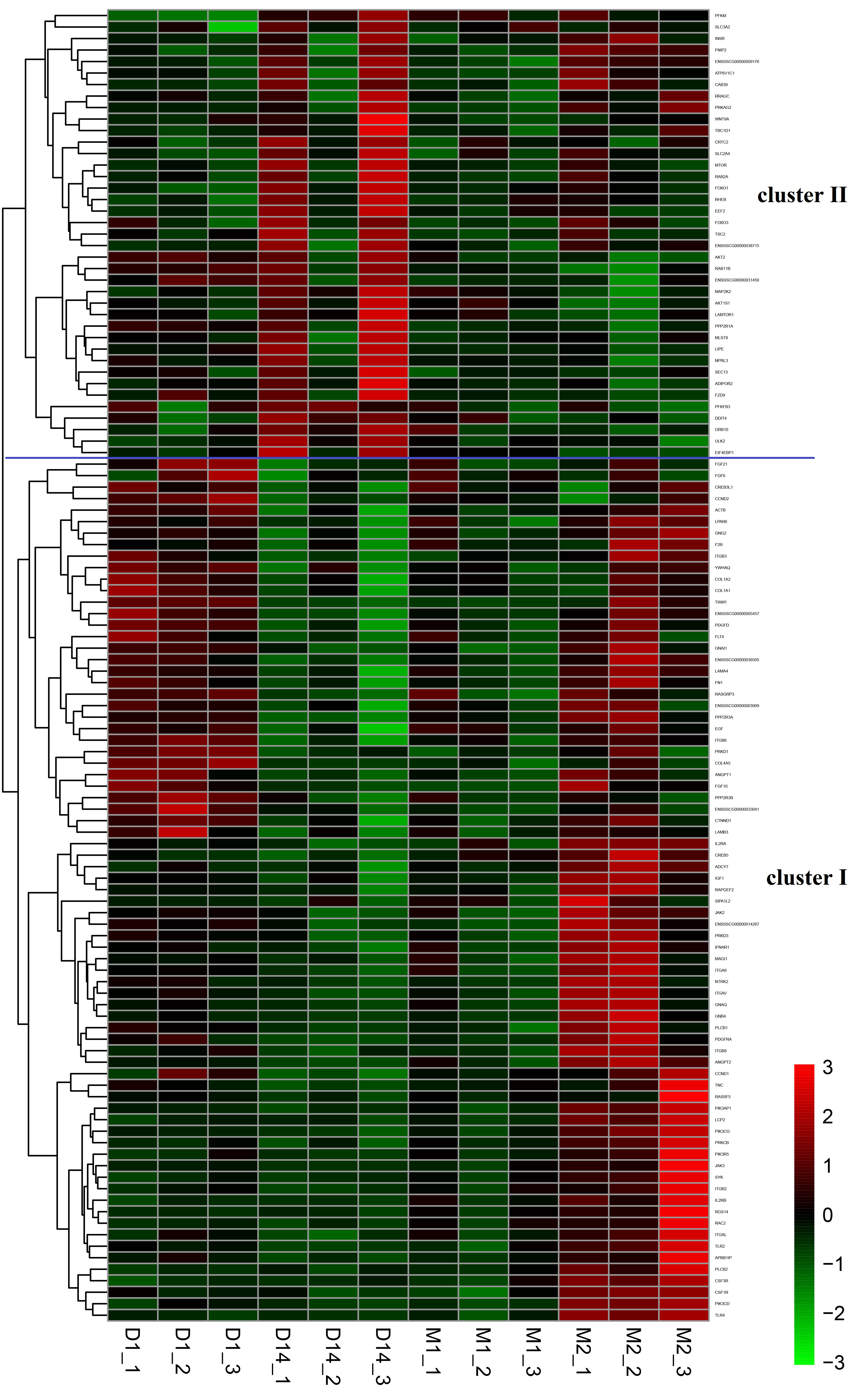

Supplement: Supplementary file 7 — Additional file 7: Fig. S1. Heatmap of the DEGs in the pathways related to muscle growth and development including Rap1 signaling, PI3K-Akt signaling, AMPK signaling, and mTOR signaling pathways. [file 12863_2021_984_MOESM7_ESM.tif]
